# Unexpected behavioural consequences of preterm newborns' clothing

**DOI:** 10.1038/srep09177

**Published:** 2015-03-17

**Authors:** Virginie Durier, Séverine Henry, Emmanuelle Martin, Nicolas Dollion, Martine Hausberger, Jacques Sizun

**Affiliations:** 1 CNRS, UMR 6552 Ethologie Animale et Humaine, Université de Rennes 1, Campus de Beaulieu, 35042 Rennes Cedex, France; 2 Université de Rennes 1, UMR 6552 Ethologie Animale et Humaine, Station Biologique de Paimpont, 35380 Paimpont, France; 3 Unité de Néonatalogie et Réanimation Pédiatrique, Pôle de la Femme, de la Mère et de l'Enfant, CHRU Brest, Avenue Foch, 29609 Brest, France

## Abstract

Restrictions of preterm newborns' movements could have consequences ranging from stress enhancement to impairment of their motor development. Therefore, ability to freely express motor activities appears crucial for their behavioural and physiological development. Our aim was to evaluate behavioural issues of two types of clothing used in NICU. We observed 18 healthy 34–37 post-conception week-old preterm newborns, during resting periods, when they were undisturbed by any interventions. Newborns wore either light clothing (bodysuit and a light wrapping) or heavy clothing (pyjamas, cardigan and sleep-sack). The percentages of time each subject spent in different postures were compared between clothing situations. Arm and hand postures differed in relation to clothing: babies bent their arms more and held their hands nearer their heads when in bodysuits than when in sleepwear. Consequently, babies in bodysuits spent more time touching their body or their environment whereas the others generally were touching nothing. Self-touch is an important way to comfort one's self. Heavy clothing may impair self-soothing behaviours of preterm newborn babies that already lack other forms of contact. Results suggest that more attention should be paid to apparently routine and marginal decisions such as choice of clothes.

Cross-cultural studies of infant development show that infants' handling routines (such as holding, bathing, dressing, and so on) could affect the onset ages of motor skills acquisitions by exposing infants to different stimulations and opportunities^1^. Among these routines, ensuring newborn babies' (NB) thermal comfort can take various forms of sleepwear ranging from mere nappies to entire body wrapping^2^. Frequently, NB in modern societies are kept in sleep-sacks that are supposed to envelop their body better and to ensure that they sleep on their back, thus lowering the risk of sudden death^3^. This is especially the case for preterm NB when they are prepared for hospital discharge. Until then, babies are in warmed incubators that prevent hypothermia^4^.

Thus, transfer from incubator to crib does not only imply a drop in NB's environmental temperature but also a drastic change of clothing, from a thin bodysuit to wearing pyjamas, a cardigan and a baby sleep-sack. This aspect may seem marginal with regard to the numerous challenges a preterm NB had had to face since birth. Yet, one can wonder whether this new situation leads to new unexpected constraints, in particular by restricting movement. At equivalent term age, former preterm NB have many skill impairments compared to full-term NB, including muscular tone patterns and quality of movements^5^,^6^. Authors hypothesized that gravity change (between in- and ex- utero life), monitoring and other medical interventions could restrict or prevent movements and thereby alter tonic responses and motor functions. The change in sleepwear and the physical constraints it involves is a typical situation that restricts or prevents movements.

The effects of movement restriction are diverse. In some psychological experiments, restraint is used to enhance stress in both human and animal subjects to evaluate their emotional responses^7^. The inability to “escape” from a situation through movement may induce increased immobility and even enhance helplessness in the long term^8^,^9^. For instance, horses that underwent a short neonatal handling procedure preventing movement showed, when exposed to novelty, locomotor inhibition rather than heightened mobility^10^. The amount of stressors perceived by very young preterm infants (assessed by Neonatal Infant Stressor Scale) also has an effect on their motor development (assessed by NICU Network Neurobehavioral Scale)^11^. Thus, the ability or not to express motor activities at this early age may be crucial for behavioural and physiological development.

Another aspect affected by physical restraint is the possibility of contact with the environment (clothes, sheets…) as well as of self-contact. It is obviously more difficult to reach one's own body parts when one's body is entirely wrapped up. Self-contact may be particularly important. Young mother-deprived primates tend either to grasp other individuals or to self-grasp^12^,^13^. Premature NB, often exposed to dys-stimulations, present early-in-life self-regulation deviations that could predict later functioning delays (i.e. delayed motor and behavioural development)^14^. Furthermore, self-contact is known to have soothing effects. For instance, light swaddling that facilitates self-contact can improve preterm NB's motor organization and self-regulatory abilities^15^.

We hypothesize that if clothing induces restraint, it may have effects on the physical and psychological well-being of NB. This study is the first step to explore this hypothesis as we assessed here the impact of two types of clothing (bodysuit vs more restraining sleepwear i.e. pyjamas, cardigan and a baby sleep-sack) on postures and behaviours of same-age preterm NB.

## Results

**Figure 1:**
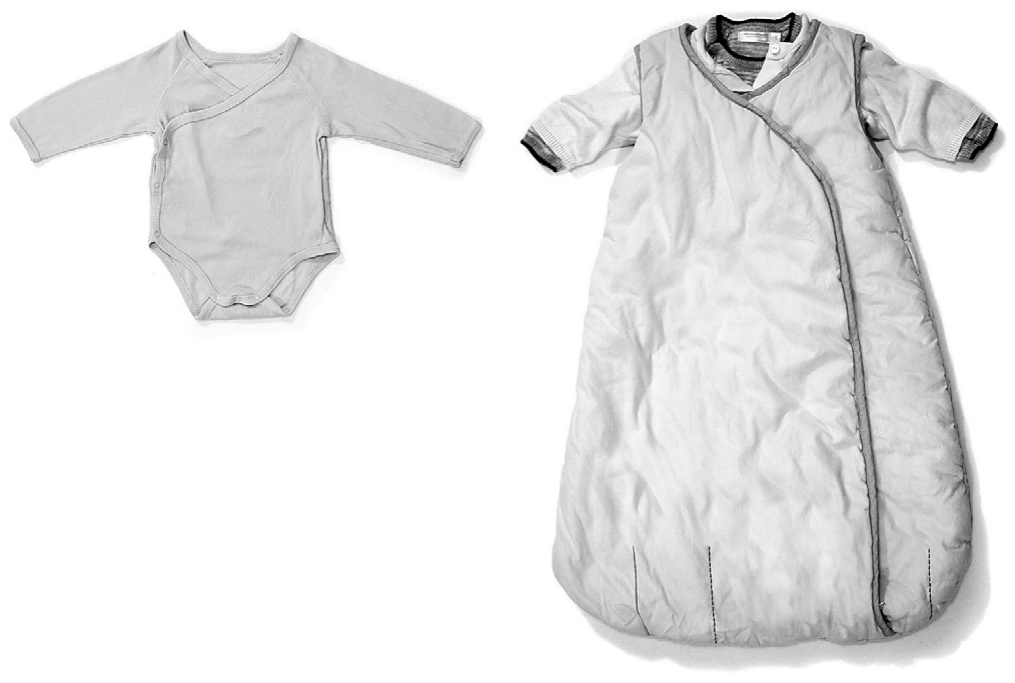
Pictures of both types of clothing. Left-hand side: a baby bodysuit; right-hand side: pyjamas covered with a cardigan and a baby sleep-sack. We thank Emmanuel de Margerie (UMR6552, Rennes, France) for providing us these pictures.

Eighteen preterm NB, aged 35 to 37 weeks post-conception, were observed while resting in a NICU, wearing either a bodysuit or a heavy sleepwear (pyjamas + cardigan + baby sleep-sack) (Fig. 1). We compared types and ranges of movements of their eyes and hands, postures of their arms and contacts between hands and surroundings between the two groups.

We evidenced significant differences between the groups of NB, indicating that clothing influenced their behaviours and postures. Thus, although all NB made few movements, when they occurred, NB in bodysuit moved indifferently one, two or three parts of their body (head, and/or arms and/or legs) whereas NB in sleepwear generally moved only one part (Friedman test: bodysuit group: X^2^ = 4.7, df = 2, P = 0.1; sleepwear group: X^2^ = 14.9, df = 2, P < 0.001, pair-wise comparisons: all comparisons P < 0.05). The arms of NB in both situations generally lay alongside their body (Friedman test: bodysuit group: X^2^ = 21, df = 3, P < 0.001; sleepwear group: X^2^ = 19.5, df = 3, P < 0.001; Mann-Whitney test: P > 0.05 for all four positions). Nevertheless, the general posture of arms differed between the two sleeping situations.

**Figure 2:**
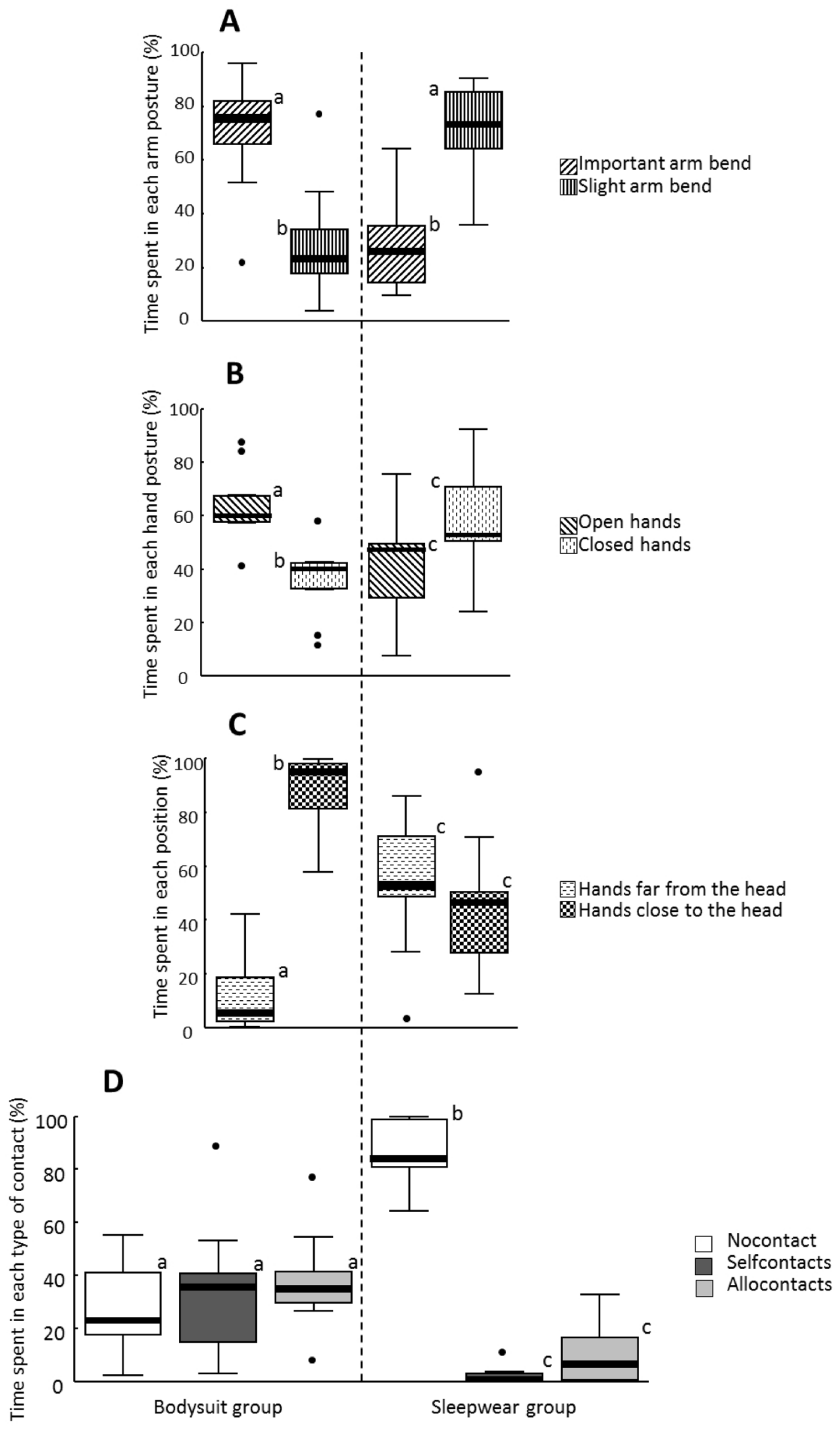
Postures of NB in bodysuit (left column) and of NB in sleepwear (right column); N = 9 NB in each group. The four postural items analysed were: A- hand posture; B- arm posture; C- hand-head proximity; D- hand contacts. The box plots represent the median (black line) and the first and third quartiles. The tails represent the minimum and maximum values, except atypical values represented as dots. Different letters refer to significantly different data (Wilcoxon tests and Friedman tests for within-group comparisons, Mann-Whitney tests for between-groups comparisons, P < 0.05).

Thus, arm-bend differed between NB in bodysuit and NB in sleepwear (Mann-Whitney test: U = 7, P = 0.002), the arms of NB in sleepwear were generally only slightly bent, and a higher proportion of the arms of NB in bodysuit presented greater arm bends (Wilcoxon test: sleepwear group: U = 43, P = 0.012; bodysuit group: U = 6, P = 0.055) (Fig. 2A).

Proximity of hands to head and hand postures differed between the two groups (Mann-Whitney test: hand proximity: U = 7, P = 0.002; hand posture: U = 16, P = 0.031): the hands of NB in bodysuit were most of the time close to their head (i.e. above chest level) and mainly open, whereas no preferred distances or postures could be evidenced for NB in sleepwear (Wilcoxon test: bodysuit group: hand proximity: U = 0, P = 0.004; hand posture: U = 3, P = 0.019; sleepwear group: hand proximity: U = 29, P = 0.49; hand posture: U = 34, P = 0.20) (Fig. 2B & C).

Consequently, hand contacts differed significantly between the two groups (Mann-Whitney test: no contact: U = 0, P < 0.001; self-contact: U = 77, P < 0.001; allocontact: U = 74, P = 0.003). Contacts of NB in sleepwear with their own body or their environment occurred rarely (respectively 0.8% [0%–11.2%] and 6.9% [0%–32.8%] of the observation time). Thus most of the time, these NB made no contact whatsoever (Friedman test: X^2^ = 14.8, df = 2, P < 0.001; pair-wise comparisons: no contact-self-contact, P = 0.006, no contact-allocontact, P = 0.006, self-contact-allocontact, P = 0.11). In contrast, NB in bodysuit made the three contact modes with similar frequencies (Friedman test: X^2^ = 0.67, df = 2, P = 0.72). Self-contacts were as frequent as contacts with the environment and represented 35.8% [2.9%–89.2%] and 35.2% [8.6%–77.5%] of the scans respectively. No contact was observed in less than a third of the scans (23.2% [2.1%–55.4%]) (Fig. 2D).

## Discussion

Comparisons of detailed behavioural observations between preterm NB wearing a bodysuit and preterm NB in sleepwear revealed significant differences in arm and hand postures. Type of clothing was associated with major differences in the amount of contact NB could have with their environment or themselves.

Overall, although all preterm NB were generally sleeping during our observations, NB in bodysuit appeared more active (more parts of body involved in movements), their arms were more flexed and their hands were open more frequently obviously seeking contact with their head (the only part of their body exposed apart from their hands). On the contrary, movements of NB in sleepwear were limited, their arm postures were tensed, they made no self-contacts with their head and their hands were closed more frequently. This particular hand posture could indicate either tenseness or a search for any type of self-contact in this situation where reaching their head may be difficult. Lifting arms towards head requires making physical efforts for which these NB may have lacked the necessary “fitness”.

Touch is the first sense to develop in utero where a foetus explores itself and its environment. This sense is available since the fourth month of pregnancy. Exploration of one's own body provides perceptual feelings that help a foetus to differentiate its body from the environment (placenta, womb walls)^16^. Double touch is a major stimulation favouring the emergence of self-concept. Indeed, when a baby or a foetus touches itself, tactile stimulations are perceived through the hand that touches a body part and through this body part touched by the hand. At birth, NB can differentiate self-touch from external stimulation^17^. In utero, foetuses seem to be able to modulate their hand movements in relation to the part of their body they are touching (eyes or mouth), and this since 22 weeks post-conception. This indicates that NB have a sense of their own body at an early age^18^. Thus, our results highlight the negative impacts that switching from light to heavy clothing could have on the development of preterm NB's self-concept.

Following premature birth, NB experience a whole new set of sensory perceptions including strong constraints on their motor capacities limiting their movements and thereby the opportunity to self-explore^19^. Furthermore, the usual NICU environment subjects preterms to intense stimulations of their immature auditory and visual systems whereas their tactile and proprioceptive systems receive minimal input^20^. Contacts received by full-term NB through skin-to-skin (or kangaroo care) procedures have a soothing effect on a short-term basis with a decrease or an absence of cries compared to physical separation from their parents (mother:^21^; father:^22^). Positive long-term effects of skin-to-skin contacts given to preterm NB have been found on different processes such as emotion regulation, stress reactivity, sleep-wake cycle or social and cognitive development^23^,^24^. These findings stress the importance of contact in such procedures. Although kangaroo care involves strong physical constraints on a baby (wrapped more or less tightly against the adult's chest), he/she perceives nevertheless a large amount of contact with his/her parent all over his/her body. With tight swaddling, temperature elevation during the hours following birth is slowed or delayed. The authors hypothesized that stress regulation could be impaired by both the absence of contact with the mother and physical restraint^2^. The constraints of the sleepwear used in this study may be similar to those of kangaroo care, but it was combined with an important lack of contact opportunities.

As mentioned above, touch plays a crucial part in young children's development of communication and of emotion regulation^25^,^26^. For instance, experiments such as the still-face paradigm evidenced an increase of self-touching during the stage when interaction with mother is impaired^27^,^28^,^29^. Self-touching is often observed associated with behaviours indicating a distressful situation (gesturing limbs, crying…) and other self-regulatory behaviours such as gaze aversion or proximity seeking^30^. Self-regulatory behaviours occur more often in emotion activating situations and are observed as young as 2 months old^27^,^31^. Infant self-touch and oral behaviours are important means of self-comfort and the younger the infant, the more touch and kinesis are important. Indeed, as their communication and perceptual skills increase, children use other behaviours to regulate their emotions and self-soothing decreases^32^,^33^.

Stressful stimulations are reduced in NICU under developmental care guidelines, but cannot be completely avoided. One recommendation is that preterm NB should be swaddled lightly, with their hands close to their face. This organisation favours sleep more than does “conventional” care^34^,^35^. As well as infants of depressed mothers, preterm NB may need self-touch to compensate from lack of touch with parents they experience due to their medical care^36^. Unfortunately, the state of sleep could not be assessed on our videos, so we are unable to determine whether sleep was impaired by type of clothing.

The postures of NB in sleepwear suggest that constraints related to this type of clothing may be stressful because it hinders movements, potentially creating a state where a baby learns that his/her attempts to move are unsuccessful (see also foals^10^), but even more it prevents soothing self-contact with his/her head. Reports suggest that physical restrictions of animals (foals) at an early stage could induce long term alterations of their emotional expression^10^,^37^. Although our study did not evaluate long term effects, it constitutes a solid basis for stimulating innovative research in this important field. Further studies should deal with this question on a long term.

These findings and questionings suggest that more attention should be paid to apparently routine issues such as the choice of clothes. For instance, consequences of the use of sleepwear on behavioural development must be assessed with various parameters of modulation in mind (such as post-natal age, gender, amount of contact received from caregivers since birth and since the use of sleepwear…). This first study must lead to further large scale developments on this issue that, if confirmed on a larger scale, should bring new information to staff and parents. Above all, special care should be given to the transfer of preterm NB from incubators where they are in light clothes to the crib where they are confronted to self-thermoregulation. The advantages and limits of the current use of sleepwear require investigation in more detail, and the relevance of our results concerning preterm NB clothing could be questioned for full-term high-risk NB.

## Methods

### Subjects

The study took place in the level II unit of the Brest University Hospital NICU. Eighteen healthy preterm NB (6 boys and 12 girls) were observed including four pairs of twins. Infants with brain injuries were excluded. Therefore, one twin was not included because of brain alterations. The characteristics of the study population are presented in table 1. The present study was approved by our institutional ethics committee and the neonates were included after their parents' informed consent had been received.

The unit followed developmental care guidelines based on the NIDCAP approach^38^. Lights and sounds were reduced as much as possible. Parents could visit their baby whenever they wanted, at any time during the night or day. Our study never disrupted care by staff or parents.

The subjects were observed in either of the two following situations (Fig. 1):

*Bodysuit group (N = 9):* each baby was in an open warmed incubator where he/she wore a bodysuit, and was loosely swaddled in a flat nappy and surrounded by a motor support device (i.e. rolled-up bed linen surrounding the baby to create “a nest”). These NB could lie either on one side or on their back.

*Sleepwear group (N = 9):* each baby was in a crib where he/she wore pyjamas and a cardigan and was placed in a baby sleep-sack. A blanket could be added over the sleep-sack. The whole set of clothes is called “sleepwear”. These NB lay exclusively on their back.

NB's characteristics, such as birth weight, birth age, weight and age when observed, did not differ significantly between the two groups (table 1). All NB were placed in an incubator following birth and were dressed in bodysuit. NB in the sleepwear group were placed in a crib as soon as the staff assessed that they were able to maintain their body temperature. Therefore, they were dressed in sleepwear. We recorded NB in the situation they were in during the short period that we could observe them. The switch from bodysuit to sleepwear had been made within a few days before the observation period.

### Observations

NB were observed during resting periods after they had been in the incubator or crib for at least 15 minutes and when no care was planned for the following half-hour. Parents could be present in the room. They could freely interact with their baby but an observation stopped as soon as they touched him/her (therefore leading to a great variety of observation durations between NB). NB were observed on two to four consecutive days, for different durations (table 1). Recordings were always made between 9 am and 5 pm on weekdays, as during the week-end family visits were more frequent and therefore resting periods were expected to be disturbed more often. For various reasons (parents slept with the NB, parents did not authorize our presence at night…), several NB could not be recorded during the night so we did not have sufficient recordings for this period for statistical analysis.

An infrared sensitive camcorder (Sony HDR-XR200) was placed on a tripod in close vicinity of the incubator or crib without hindering access to the baby by parents or staff. The camcorder was mainly used with the night-shot mode on because the rooms were dimly lit. The baby's whole body was filmed.

### Video analysis

As we chose to film the infants' entire body, we could not analyze their facial features in detail. We focused mainly on postures and contacts. The sampling method was the instantaneous scan sampling^39^, i.e. we recorded the behavioural and postural items expressed by each baby at 30-second intervals (table 2).

Whatever the situation, NB mostly slept on their back during our observations (bodysuit group: 80.6% [10.8%–100%]; sleepwear group: 81.7% [38.5%–100%]). We decided to focus our comparisons on the constraints due to clothing, not the general sleeping position. Therefore, we compared the items recorded only when NB were on their back.

As we recorded NB during resting periods, their eyes were generally closed whatever their clothing (bodysuit group: 93.4% [5.3%–100%]; sleepwear group: 93.5% [71.1%–98.8%]; Mann Whitney U test: P > 0.05 for all eye modalities). Therefore, eye open was not included in further tests.

### Statistical analyses

We detailed and evaluated the postures and behaviours of NB from the start of the recording until someone touched or spoke to them. Thus these interruptions caused important interindividual variations in the durations of recorded time suitable for analysis (table 1). Therefore, we did not compare raw numbers of occurrences (scans) but we compared percentages of scans presenting each item. Percentages cited in the text represent the median [range] of the data set.

Data were analysed with non-parametric statistics, using R. All tests were two-tailed. When data could be obtained for an item concerning both sides of the NB (hand posture for instance), we investigated side effect. As none could be evidenced (Wilcoxon tests, P > 0.05 for all comparisons), data for both sides were pooled for further analyses.

Within group comparisons evaluated the relative importance of each behavioural and postural modality for each item. Wilcoxon tests and Friedman tests followed by pair-wise comparison tests with Benjamini and Hochberg adjustment method^40^ were applied for intra-group comparisons. Inter-group comparisons evaluated the influence of sleeping situations on NB's behaviour and/or postures (Mann-Whitney test).

### Ethics statement

The current study was carried out in accordance with the approved guidelines of our institutional ethics committee (CHU Brest). Parents of neonates gave written informed consent before the NB were included in the study.

## Author Contributions

V.D., S.H., M.H. & J.S. designed the experiment, J.S. organized the population recruitment, V.D., E.M. and N.D. collected the video database and performed the analyses, S.H. and M.H. contributed to the statistical analysis, V.D., M.H., S.H. and J.S. wrote the manuscript, J.S. and M.H. contributed equally.

## Figures and Tables

**Table 1:** Population characteristics and statistical differences between the two studied groups

	Bodysuit group	Sleepwear group	Mann-Whitney tests
*NB characteristics*			
Birth gestational age (weeks + days)	30 + 3 [27–33 + 5]	30 + 3 [27 + 4–32 + 4]	U = 35, P = 0.66
Birth weight (grams)	1460 [935–2165]	1425 [1035–1680]	U = 27, P = 0.8
Post-natal age (days) when observed	34.5 [11–59,5]	34.5 [22–69]	U = 40, P = 1
Weight when observed	1864 [1415–2303]	2020 [1605–2304]	U = 20, P = 0.08
*Analyzed videos*			
Duration (minutes) per baby	141 [88,5–264]	163.5 [60.5–343]	U = 28, P = 0.29
Duration (min) on their back^a^	107 [14–233]	125.5 [48.5–343]	U = 28, P = 0.29

a: only videos of NB lying on their back were included in the analyses

Bodysuit group, N = 9; Sleepwear group, N = 9 (see text for group description)

**Table 2:** Observed behavioural and postural items and their different modalities

ITEMS	DEFINITIONS
Movement type	Scored: 0 to 3 according to the number of body parts moving. They could be either the head or at least one hand or at least one leg (visible when the bottom of the sleep-sack moved).
	- 0 = no movement
	- 1 = one body-part involved
	- 2 = two body-parts involved
	- 3 = three body-parts involved
Movement range	Scored: small/medium/high according to the range of the movement
	- small = brief movement like a shudder, the general posture was not affected
	- medium = the body part moved less than 45° from its initial position
	- high = the body part moved over 45° from its initial position
Head orientation	Scored: left/front/right according to the position of the head
Body orientation	Scored: left/back/right according to the position of the body (except the head)
Eyes	Scored: closed/half-open/open
	- closed = both eyes closed
	- half-open = upper eyelid of one eye covering more than half the eye, the other eye could be closed or half-open
	- open = upper eyelid of one eye covering less than half the eye, the other eye could be closed, half-open or open.
For each arm:	
Angle	8 positions were scored: 1 to 4 according to the angle between the arm and the chest combined with T or A according to the position of the arm in relation to the body
	- 1 = arm between 0° and 45° from the chest (arm alongside body)
	- 2 = arm between 45 and 90°
	- 3 = arm between 90° and 135°
	- 4 = arm between 135° and 180° (arm close to the head)
	- T = arm above the body (Toward the central axis of the body);
	- A = arm sideways (Away from the central axis of the body)
	example: 1A = arm oriented away and between 0° and 45° from the body axis
Bend	Scored: + or – according to the angle between the arm and the forearm
	+ = when the bend was important (angle less than 90°)
	- = when the bend was slight (angle above 90°)
Hand proximity	Scored: close/far according to the proximity of hand to head
	- close = hand above a virtual line level with chest
	- far = under a virtual line level with chest
Hand posture	Scored: closed/open
	- closed = all fingers were folded
	- open = at least one finger was unfolded
Hand contact	Scored: no contact/self-contact/allocontact
	- no contact = neither the palm nor the back of the hand was touching something
	- self-contact = either the palm or the back of the hand was touching part of the body, mainly the other hand, face or head
	- allocontact = either the palm or the back of the hand was touching something around the infant while the other side touched nothing
